# Tribological Properties of AlSi12-Al_2_O_3_ Interpenetrating Composite Layers in Comparison with Unreinforced Matrix Alloy

**DOI:** 10.3390/ma10091045

**Published:** 2017-09-06

**Authors:** Anna Janina Dolata

**Affiliations:** Faculty of Materials Engineering and Metallurgy, Silesian University of Technology, Krasińskiego 8, 40-019 Katowice, Poland; anna.dolata@polsl.pl; Tel.: +48-32-603-4426

**Keywords:** aluminum matrix composites (AMCs), interpenetrating composites (IPCs), alumina foams, centrifugal infiltration, tribological properties

## Abstract

Alumina–Aluminum composites with interpenetrating network structures are a new class of advanced materials with potentially better properties than composites reinforced by particles or fibers. Local casting reinforcement was proposed to take into account problems with the machinability of this type of materials and the shaping of the finished products. The centrifugal infiltration process fabricated composite castings in the form of locally reinforced shafts. The main objective of the research presented in this work was to compare the tribological properties (friction coefficient, wear resistance) of AlSi12/Al_2_O_3_ interpenetrating composite layers with unreinforced AlSi12 matrix areas. Profilometric tests enabled both quantitative and qualitative analyses of the wear trace that formed on investigated surfaces. It has been shown that interpenetrating composite layers are characterized by lower and more stable coefficients of friction (μ), as well as higher wear resistance than unreinforced matrix areas. At the present stage, the study confirmed that the tribological properties of the composite layers depend on the spatial structure of the ceramic reinforcement, and primarily the volume and size of alumina foam cells.

## 1. Introduction

The new directions of research in the area of metal matrix composites (MMCs) include the activities aimed at the development of effective methods for manufacturing these materials. At present, the composites with layered or gradient structures [1,2,3,4,5,6], multiphase composites (hybrid, heterophase) [7,8,9,10,11], and particularly ceramic–metal interpenetrating composites (IPCs) are investigated [12,13,14,15,16,17]. Special emphasis is placed on the development of “net shape” or “near net shape” technologies, which to a large extent allow the elimination or reduction of the machining of composite products, and thus reduce both wastes and production costs. Casting aluminum alloys are most often used for matrix composites due to their advantageous properties, low price, and density [18]. In turn, by the proper selection of the type, size, volume fraction, and morphology of the reinforcing ceramic components, as well as the composite manufacturing method, it is possible to produce aluminum matrix products with special properties.

It has been noted that the interpenetrating composites (IPCs), which consist of 3-dimensionally continuous matrices of two different phases (ceramic and metallic), are interesting materials with potentially superior properties when compared with traditional composites containing discontinuous particles or whiskers as well as continuous or short fibers [14,19,20,21]. For example, Peng et al. [19] reported that alumina–aluminum interpenetrating phase composites show a higher modulus of elasticity compared with the traditional AA6061/Al_2_O_3_ Duralcan composites. Similarly, the results of our own previous studies [22,23] showed that the hardness, compressive strength, and Young’s modulus in such composites increase, while decreasing the pores’ size in alumina foam. It has been clearly demonstrated that the level of any IPC’s properties depends on the degree of filling empty ceramic spaces by the liquid Al alloy. The favorable mechanical characteristics are the result of a specific composite macrostructure with percolation of ceramic and metal phases. 

Due to the potential applications for IPCs in the machine, construction, and automotive industries (disc brake, pistons, cylinder sleeve, etc.), good mechanical properties such as thermal and dimensional stability and proper wear resistance are essential. The research conducted by Binner et al. [20,21] showed that the Al(Mg)/Al_2_O_3_ interpenetrating composites obtained by pressureless infiltration have significantly better wear resistance than matrix alloy, and moreover in comparison to conventional AA6061/Al_2_O_3_ and AA2014/Al_2_O_3_ Duralcan composites. The authors found that the composite made from the lowest foam density exhibited a ‘ploughing’ wear throughout the process, whilst the composites with the higher foam density, and hence a higher hardness and load-bearing capability, exhibited a transition from ‘ploughing’ to ‘protective’ wear [20]. The obtained results are promising for the potential use of these new engineering materials in areas requiring wear resistance coupled with lightweight applications. However, it should be noted that in the process of designing the composition and structure of a composite material intended for tribological cooperation, external factors enforcing a certain set of the material reaction are taken into consideration. These include: load, operational temperature, lubrication type, speed of movement, presence of vibration. They also include a broad range of structural properties of the material, such as the type of matrix and the reinforcing phase, the fraction and size of the reinforcing phase, and its morphology [24,25,26]. Each of these factors has a direct influence on the durability and reliability of a tribological pair. Changing any from these factors can give different results, which justifies further research in this area.

In our own previous papers [27,28,29,30], the theoretical background, results of experiments and structure of AlSi12/Al_2_O_3_ interpenetrating composite layers obtained by the centrifugal infiltration method, have been described in detail. The aim of research presented in this work was to compare the tribological properties (wear resistance, friction coefficient) of these composites with unreinforced AlSi12 matrix alloy. Profilometric tests enabled quantitative and qualitative analyses of the wear trace that formed on investigated surfaces.

## 2. Materials and Methods

**Figure 1:**
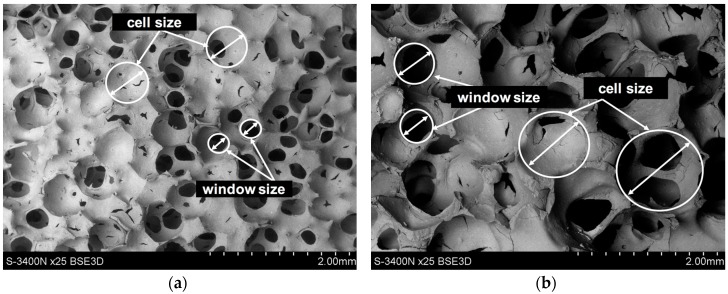
Scanning electron microscopy (SEM) micrographs of alumina oxide preforms used for centrifugal infiltration process by liquid AlSi alloy: (**a**) Al_2_O_3__1; (**b**) Al_2_O_3__2.

**Figure 2:**
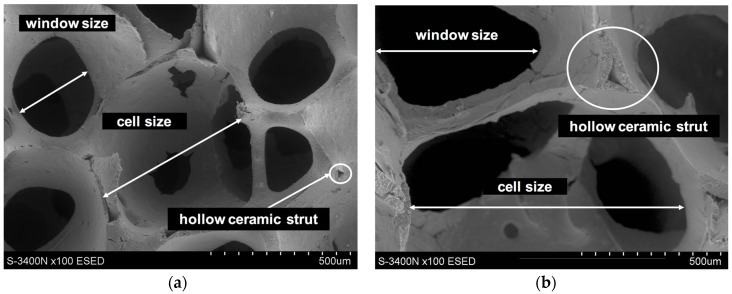
SEM micrographs of alumina oxide preforms used for centrifugal infiltration process by liquid AlSi alloy: (**a**) Al_2_O_3__1; (**b**) Al_2_O_3__2.

**Figure 3:**
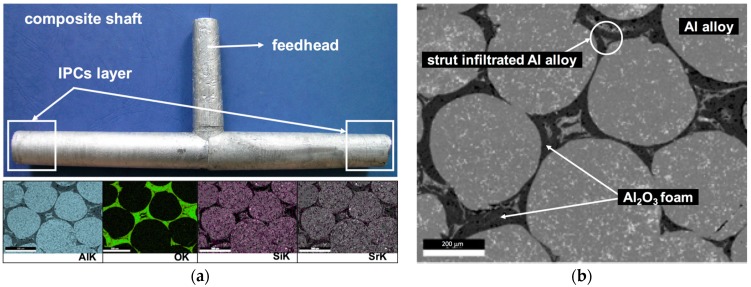
The AlSi12/Al_2_O_3__2 composite cast obtained by centrifugal infiltration: (**a**) view of the representative centrifugal cast in the form of locally reinforced shaft, and X-ray mapping of Al, O, Si and Sr in area of reinforcement; (**b**) SEM image of interpenetrating composites (IPCs) layer.

The alumina oxide foams (Al_2_O_3_) with various total porosity and different cell size (Figure 1 and Figure 2) resulting from the applied manufacturing method (replacement of porous polymer matrix) were used as reinforcing [28,30]. In the first foam, which was designated as Al_2_O_3__1, slight differences of cell size were observed (Figure 1a and Figure 2a). Over 60% were in the range from 350 to 550 μm. The second foam, Al_2_O_3__2, is characterized by larger pore diameters and a much greater dispersion of their size from 300 to 1150 μm, where over 50% of cell sizes were in the range of 800–1150 μm (Figure 1b and Figure 2b). To shape the castings, locally reinforced via ceramic skeletons with known spatial structures, and the centrifugal infiltration process were used [27,28,29,30]. As a result of the centrifugal force acting on the liquid AlSi12 alloy surface, castings containing a composite layer with percolation structure were obtained (Figure 3). The residual porosity of the reinforced areas, measured by computer-assisted tomography, did not exceed 1% [30]. Moreover, the complete filling of the cells, and the absence of structure defects and discontinuities at the metal–ceramics interface, have been confirmed by detailed examinations using scanning electron microscopy and energy dispersive spectroscopy (SEM, EDS), as described in previous works [27,30].

Tribological studies (wear resistance, coefficient of friction) were carried out in cross-sections of the composite layer formed by the centrifugal infiltration process and compared with the unreinforced area. The samples used to determine the tribological properties (Table 1), sized 30 × 15 × 10 mm, were cut out from composite shafts and polished before testing. Thus, a prepared composite and matrix surface was subjected to the abrasion test under dry sliding conditions using tribology pin-on-block tester [31]. A normal load of 15 N (unit pressure of 2 MPa) and a sliding speed of 0.1 m/s were applied throughout the tests. The counter-pin material, ϕ = 3 mm and 20 mm in length, was made of EN-GJ250 cast iron. The tests were carried out with the 9-mm stroke length over a distance of 1000 m at air temperature (20 °C). During tests, the friction coefficient was measured. The obtained results were presented in the form of graphs as a function of the sliding distance. Identical load conditions and abrasion speed allowed the comparison of friction coefficient values and the wear of composites with those of the unreinforced matrix. 

The wear trace that appeared on the surface of composite and matrix samples was subjected to profilometric analyses using the MicroProf 3000, FRT optical profilometer (FRT GmbH, Germany). The study of the wear trace geometry was carried out immediately after the friction process. Only an ultrasonic scrubber was used to clean the surface of the tested samples. The basic features of the surface, such as the depth of the wear trace and roughness, were assessed. 2D and three-dimensional images were used in the analysis. The wear resistance of the tested samples (AlSi12 matrix and AlSi12/Al_2_O_3_ composite layers) was determined based on volume loss measurements of the wear traces formed on their surfaces. The research was carried out based on 3D image analysis with 0.1 μm accuracy in *X*- and *Y*-axes, and with 0.01 μm in *Z*-axis.

## 3. Results and Discussion

**Figure 4:**
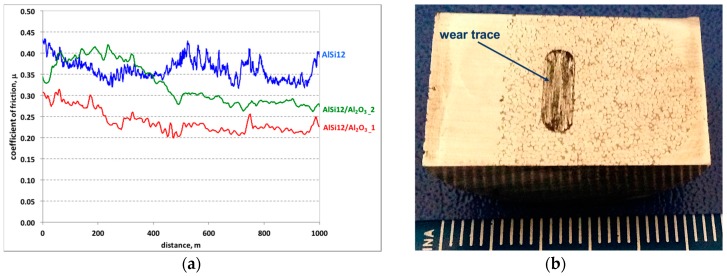
Friction coefficient (μ) versus sliding distance for un-reinforced area (AlSi12 matrix alloy) and AlSi12/Al_2_O_3_ foam composite layers (**a**); view of AlSi12/Al_2_O_3__1 composite surface after dry sliding wear test (**b**).

**Figure 5:**
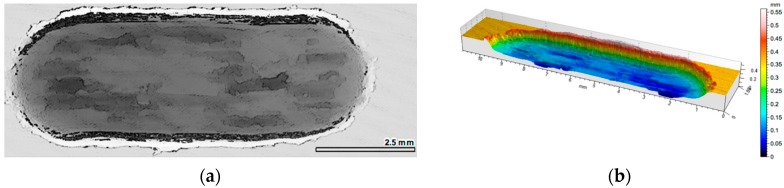
View of wear track in unreinforced area (AlSi12 matrix) after dry sliding condition: (**a**) digital image; (**b**) cross-section through 3D view.

The results of the friction coefficient measurement as a function of the sliding distance for the matrix area (without reinforcement), and comparisons with areas reinforced by two different alumina foams, are shown in Figure 4a. It was observed that in the case of unreinforced areas (AlSi12 matrix alloy), the course of changes in the coefficient of friction is unstable, with variations of 0.1. Such sudden changes in friction coefficient values are characteristic for the adhesive wear mechanism, which is also confirmed by profilometric observations of the wear track surface (Figure 5).

**Figure 6:**
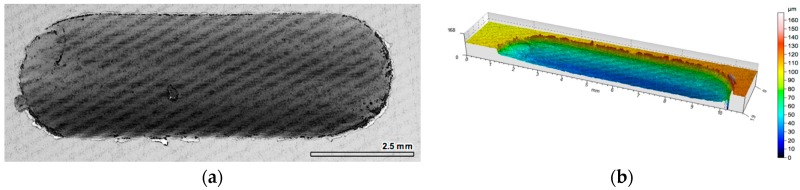
View of wear track in AlSi12/Al_2_O_3__1 composite layer (P1) after dry sliding condition: (**a**) digital image; (**b**) cross-section through 3D view.

**Figure 7:**
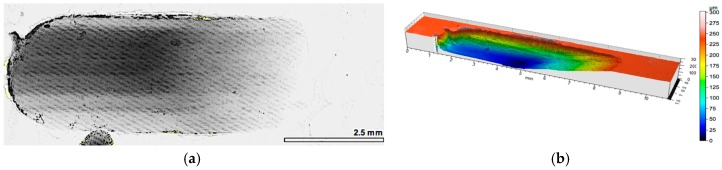
View of wear track in AlSi12/Al_2_O_3__2 composite layer (P2) after dry sliding condition: (**a**) digital image; (**b**) cross-section through 3D view.

In turn for both composite layers with a percolation structure (red and green lines in Figure 4a), which differ in porosity, and cell size in alumina foam, a similar character of friction coefficient change was recorded. At the initial stage of friction (500 m), the coefficient of friction is significantly higher than its value recorded in the second half of the sliding distance. For the composite layer marked P1, the value of the friction coefficient during the sliding distance changes from μ = 0.3 to μ = 0.23, while for composite layer signed P2, this change is μ = 0.4 in the first part, and μ = 0.27 at the final stage of friction, respectively. The 2D and 3D images of the wear track of the composite layers have been shown in Figure 6 and Figure 7.

As can be seen, the friction coefficient of the tested composite layers decreases as the pores diameter in the alumina foam decreases. In both cases, following the running-in phase (about 500 m), the friction coefficient stabilized, and remained stable until the end of the test. Such a characteristic course of changes in the coefficient of friction could be related to the change of wear mechanism from adhesive—abrasive to the abrasive only. As can be expected, the stabilization of the friction coefficient obtained by alumina foam addition will have a positive effect on the operation of the tribological system’s cast iron—composite layer.

**Figure 8:**
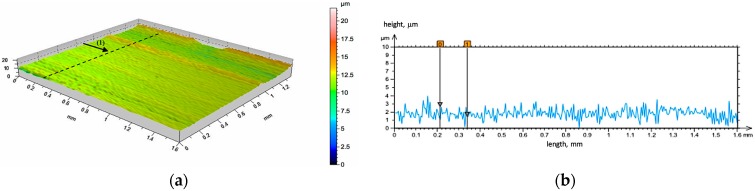
Initial state of the surface geometry of the unreinforced area: (**a**) 3D view; (**b**) height difference on line (1).

**Figure 9:**
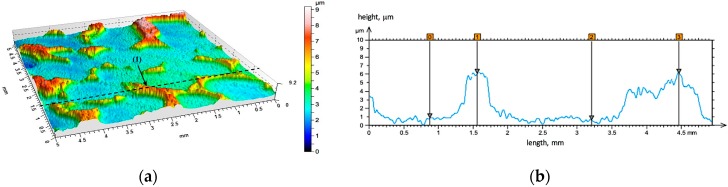
Initial state of surface geometry of the AlSi12/Al_2_O_3__2 composite layer (P2): (**a**) 3D view; (**b**) height difference on line (1).

**Figure 10:**
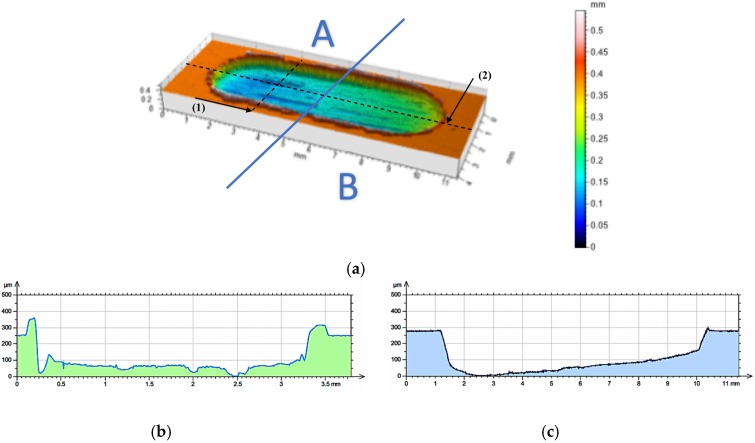
Surface geometry of the AlSi12 unreinforced area after working with cast iron pin: (**a**) view of the wear track; (**b**) roughness distribution across to the friction direction on line (1); (**c**) roughness distribution along the friction direction on line (2).

**Figure 11:**
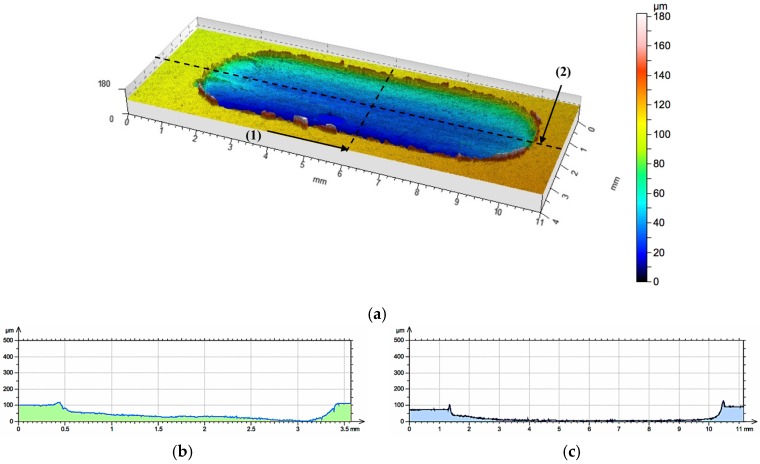
The surface geometry of the AlSi12/Al_2_O_3__1 composite layer (P1) after working with cast iron pin: (**a**) view of wear track; (**b**) roughness distribution across to the friction direction on line (1); (**c**) roughness distribution along the friction direction on line (2).

**Figure 12:**
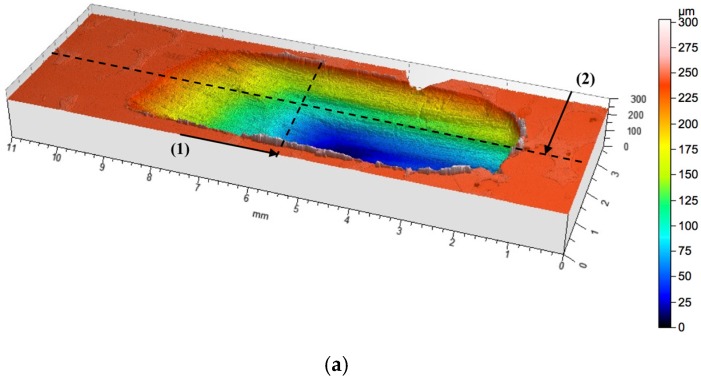

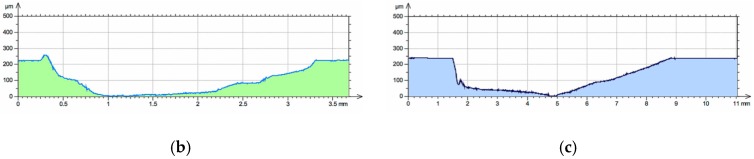
Surface geometry of AlSi12/Al_2_O_3__2 composite layer (P2) area after working with the cast iron pin: (**a**) view of the wear track; (**b**) roughness distribution across to the friction direction on line (1); (**c**) roughness distribution along the friction direction on line (2).

In addition, a detailed analysis of the tested materials surface both before and after friction were performed based on the profilometric measurements. The initial state of the surface geometry of the unreinforced area (matrix AlSi12 alloy) and AlSi12/Al_2_O_3__2 composite layer (P2) is shown in the Figure 8, Figure 9 and Figure 10, respectively. As can be seen from the presented images, the surface of the composite layers has different a geometry in comparison with unreinforced areas. The distribution of the ceramic cells is regular, and their shape is clearly extended (Figure 9a). In this case, as well as for the second tested composite layer (P1), the ceramic reinforcement protruding above the matrix at a height of about 5 μm was observed (Figure 9b—exemplary line marked on Figure 9a). Future profilometric tests enabled quantitative and qualitative analyses of the wear trace that formed on investigated surfaces (Figure 10, Figure 11 and Figure 12).

The profilometric analysis of the AlSi12 matrix wear track images confirmed the specific, plastic material deformation, 0.4 mm in depth, formed at the edges (Figure 10). 

The quantitative analysis performed for the AlSi12 matrix wear track surface after dry friction conditions showed intensive wear. On the basis of qualitative observation, the wear trace can be divided into two zones (Figure 10a). In the first area, marked A, the deep wear with abrasive phenomenon can be observed. In the second zone, determined as B, the wear has an adhesive character. In turn, the depth of the wear track calculated as the maximum difference of elevation of the wavy line and its lowest position, in the case of AlSi12 matrix area after cooperation with cast iron pin, is 0.25 mm.

The use of local reinforcement in the form of ceramic alumina foams has resulted in a significant increase in the wear resistance under technically dry friction conditions. In both cases, the consumption was based on abrasive wear, irrespective of the cell size and the volume fraction of the ceramic phase (Figure 11 and Figure 12). For the P1 composite layer (Figure 11), the depth of the wear track did not exceed 100 μm. In turn, the P2 composite (Figure 12) was characterized by a twice as large depth of the wear track, which reaches 220 μm. However, in both materials, plastic deformation was not observed, and matrix elevation at the wear track edges did not exceed 20 μm in depth.

**Figure 13:**
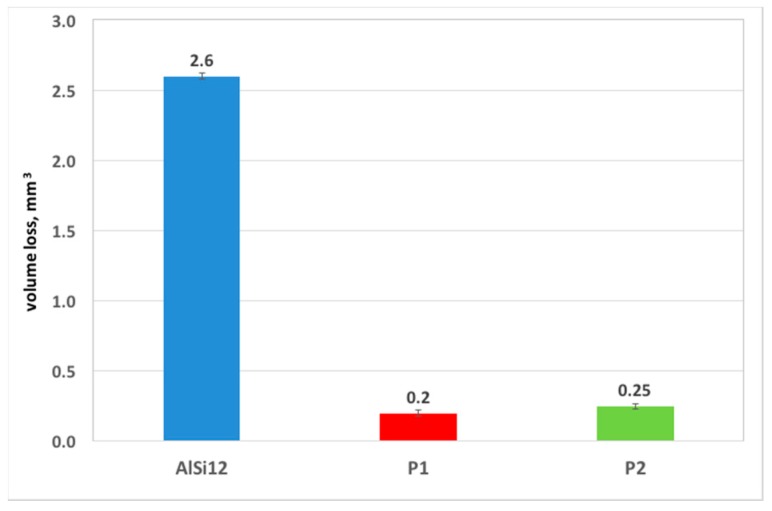
Volume loss of investigated interpenetrating composite layers (P1 and P2) in comparison with the unreinforced matrix area (AlSi12) in dry sliding conditions.

Furthermore, the volume loss of the wear traces was measured in order to compare the wear resistance of the examined reinforced and unreinforced areas. The obtained results (Figure 13) clearly showed a 10 times smaller volume loss of composite layers compared with the unreinforced matrix area in dry friction conditions.

The lower wear in composite layers compared with the unreinforced matrix can be attributed to an Al_2_O_3_ network protruding out of the worn surface, which protects the direct wear of the AlSi12 matrix alloy by the cast iron pin.

## 4. Conclusions 

The interpenetrating composite layers obtained in the centrifugal infiltration process have a good connection at the interface between alumina preforms and the AlSi12 matrix. The strong boundary and the characteristic interpenetrating structure of the composite layers influence tribological properties (wear resistance, friction coefficient). The investigation results proved that the composite layers with the Al_2_O_3_ foams are characterized by a lower friction coefficient of about 30% for P1 foam and near 25% for P2 in comparison with the unreinforced area in the cast. Moreover, it has been shown that the friction coefficient of composite layers decreases as the pores’ diameters decrease. In turn, the composite layers’ higher wear resistance in comparison with matrix areas is related to the change of wear mechanism from adhesive—abrasive to the abrasive only. 

In addition, it has been proved that local reinforcement of castings improves their properties only in areas that are highly exposed to wear, and it allows to maintain the initial mechanical and plastic properties in unreinforced areas. This solution is favorable, particularly from the point of view of the finishing problems of composite products.

Further studies will concern a wider description of the wear mechanism of IPCs in various friction coupling.

## Figures and Tables

**Table 1:** Designation of samples used to determine tribological properties.

Designation	Material	Volume of Al_2_O_3_ [%]	Pore size [μm]
P0	AlSi12 matrix	-	-
P1	AlSi12/Al_2_O_3__1	22	350–550
P2	AlSi12/Al_2_O_3__2	18	300–1100
